# Increased Resolvin E1 Production by Peripheral Blood Mononuclear Cells in Periodontitis Patients: Pilot Study

**DOI:** 10.1590/0103-6440202405861

**Published:** 2024-10-25

**Authors:** Lina J. Suárez, Wilmer González-Duarte, Rodrigo Torrez-Velasco, Viviana Salinas, Nelly S. Roa-Molina, Sindy M. Muñoz, Luz-Stella Rodríguez, Roger M. Arce, Jamil A. Shibli, Adriana Rodríguez-Ciodaro

**Affiliations:** 1 Universidad Nacional de Colombia, Departamento de ciencias básicas y medicina oral, Facultad de Odontología, Bogotá, Colombia.; 2 Pontificia Universidad Javeriana, Centro de investigaciones odontológicas, Facultad de Odontología, Bogotá, Colombia.; 3 Pontificia Universidad Javeriana, Instituto de genética Humana, Facultad de Medicina, Bogotá, Colombia.; 4 Department of Periodontics and Dental Hygiene, School of Dentistry, University of Texas Health Science Center at Houston, Houston, TX, United States.; 5 Guarulhos University, Department of Periodontology, Dental Research Division, Guarulhos, Brasil.

**Keywords:** Leukocytes Mononuclear cells, Periodontitis, Pro-resolving lipid mediators, Trained innate immunity

## Abstract

This study quantified the production of the pro-resolving agent Resolvin E1 by peripheral blood mononuclear cells (PBMC) from 20 systemically healthy volunteers with and without periodontitis after stimulation with lipopolysaccharide (LPS) from Porphyromonas gingivalis (Pg). Ten periodontitis patients and 10 healthy volunteers (30-50 years old), matched by age and sex, were recruited. Peripheral blood mononuclear cells were isolated and stimulated in culture plates for 24 hours with Pg LPS. Resolvin E1 levels were measured in the supernatants by enzyme-linked immunosorbent assay. Significantly higher production of Resolvin E1 was observed in both groups when stimulated with LPS compared to baseline levels (p<0.001). A significant increase in Resolvin E1 was observed in the presence of Lipopolysaccharide in the patients with periodontitis compared to the healthy group (p=0.0019). Resolvin E1 levels may reflect a measure of resolution of inflammation that warrants further clinical investigation.

## Resumo

Este estudo quantificou a produção do agente pró-resolução Resolvin E1 por células mononucleares de sangue periférica (PBMC) de 20 voluntários sistemicamente saudáveis com e sem periodontite após a estimulação com lipopolissacárido (LPS) de Porphyromonas gingivalis (Pg). Se reclutaram dez pacientes com periodontite e 10 voluntários saudáveis (30-50 anos), emparejados por pai e sexo. Células mononucleares de sangue são isoladas e estimuladas em placas de cultivo durante 24 horas com Pg LPS. Os níveis de Resolvin E1 são mediados nos sobrenadantes por meio de um ensaio imunoabsorvente ligado a enzimas. Foi observada uma produção significativamente maior de Resolvin E1 em ambos os grupos quando houve estímulo com LPS em comparação com os níveis iniciais (p=0,001). Foi observado um aumento significativo de Resolvina E1 na presença de lipopolissacárido em pacientes com periodontite em comparação com o grupo saudável (p=0,0019). Os níveis de Resolvin E1 podem refletir uma medida de resolução da inflamação que justifica uma investigação clínica aprofundada

**Figure:**
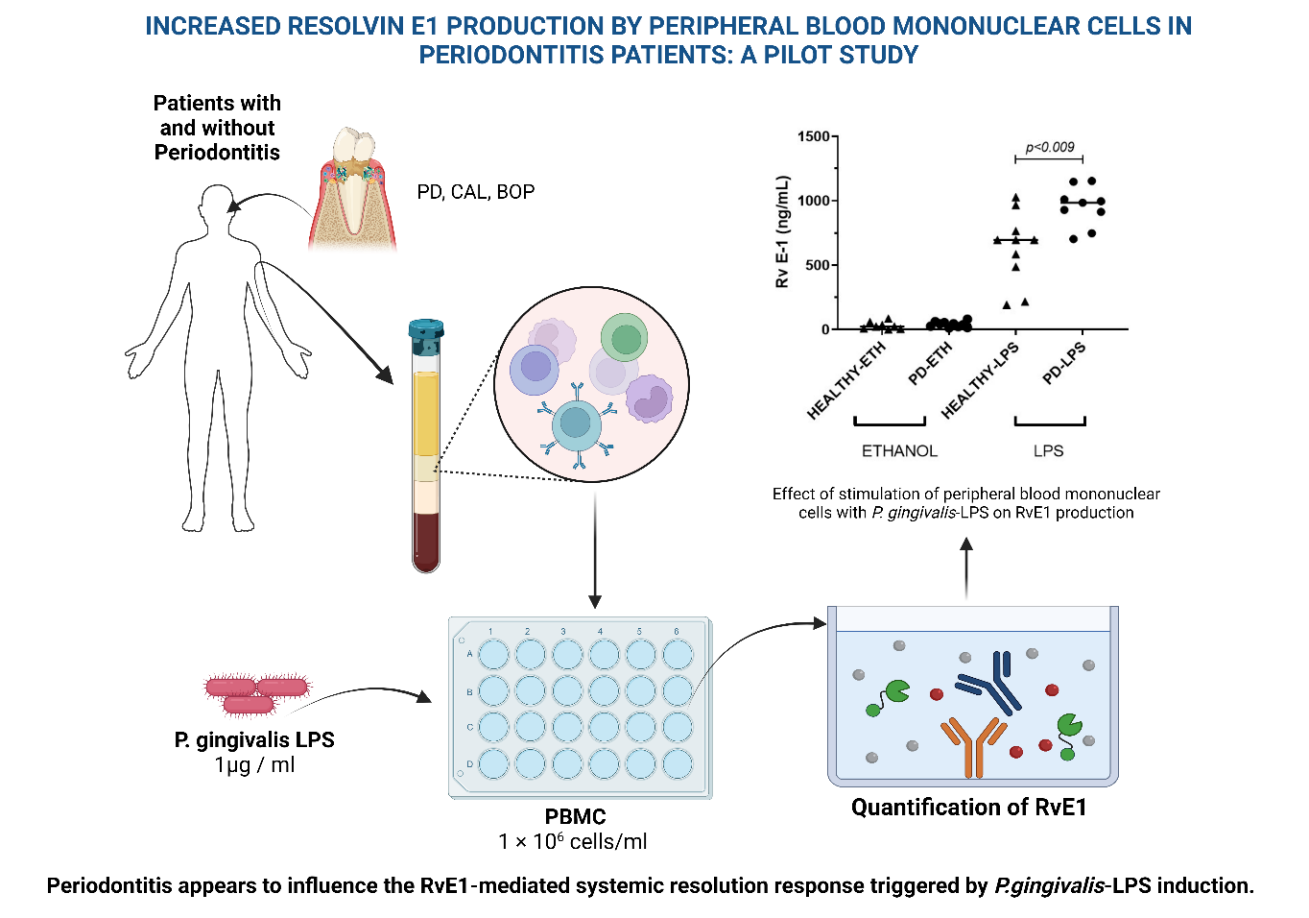

## Introduction

Local damage observed in periodontitis is a result of the dysregulated activation of the inflammatory process, which is initiated by bacterial dysbiosis in the oral cavity^1^. This local pathogenic process, in response to continuous bacteremia, not only leads to tissue damage but also generates low-magnitude systemic inflammation^2^ ^,^ ^3^.

Research on the inflammatory response to infectious agents has explored the potential for self-resolution achieved by the organism through pro-resolving mediators. These mediators are produced through the enzymatic conversion of acids and essential fats, and they function by reprogramming the immune response to facilitate the elimination of invading pathogens and counter-regulate the production of inflammation-initiating molecules. This process promotes tissue repair and regeneration, playing a crucial role in reestablishing barriers and preventing reinfection^4^.

Among these agents, resolvins have been characterized as pro-resolving and anti-inflammatory molecules. Resolvins are known to mitigate tissue damage and stabilize the host's inflammatory response without suppressing the immune system. One notable subclass of resolvins is resolvin E1 (RvE1), which is derived from omega-3 eicosapentaenoic acid. RvE1 is attributed to reducing the infiltration of inflammatory cells, promoting the phagocytosis of apoptotic cells, as well as activating the transcription factor NF-κB^4^ ^,^ ^5^.

In the context of periodontal disease, studies conducted in animal and human models have demonstrated that the application of this molecule or a significant increase in its local production through dietary supplements can influence clinical markers of periodontal disease. These effects may include reductions in pocket depth, bone loss, and the regeneration of periodontal tissues ^2^ ^,^ ^6^ ^,^ ^7^.

Beyond its clinical impact, RvE1 has been observed to bind specifically to neutrophils through the recombinant ChemR23 receptor. This activity inhibits the formation of superoxide anions in patients with aggressive localized periodontitis^8^.

While the pathways through which pro-resolutive effects occur and their potential to resolve chronic inflammatory processes have been identified, the plasma levels of efficient pro-resolving agents like RvE1 have yet to be comprehensively characterized^4^ ^,^ ^9^. Assessing the systemic response after local inflammation typically involves examining the expression of pro-inflammatory factors and acute phase proteins; however, there is limited data regarding how this systemic response is regulated and resolved in both healthy and diseased states. Furthermore, the resolution of the systemic response in the face of a constant acute bacterial challenge remains unclear, despite the well-established link between dysbiosis and atopobiosis of the periodontal microbiome and the maintenance of systemic micro-inflammation^9^.

Drawing from our understanding of failures in resolving the inflammatory response in various human diseases (including periodontal diseases), we conducted a pilot evaluation of systemic pro-resolving behavior in individuals with localized periodontitis. Therefore, this study quantified the production of RvE1 by peripheral blood mononuclear cells (PBMC) in systemically healthy volunteers, both with and without periodontal disease, following activation with lipopolysaccharide (LPS) derived from *Porphyromonas gingivalis (Pg)*.

## Material and methods

### Study population

20 individuals attending the Postgraduate Periodontics Clinic at the Universidad Nacional de Colombia were recruited and voluntarily consented to participate in the study. Study subjects were divided into 2 groups: periodontitis patients (n = 10) and healthy controls (n = 10) (matched by sex and age).

The participants' ages ranged from 30 to 50 years, and they were required to have a minimum of 18 teeth. Exclusion criteria encompassed systemic diseases, including cancer, immunodeficiency syndromes, metabolic bone diseases, disorders affecting wound healing, radiation exposure, or immunosuppressive therapy. Other exclusion criteria comprised smoking, pregnancy, lactation, recent systemic antibiotic treatment within the preceding 2 months, chronic use of non-steroidal anti-inflammatory drugs (NSAIDs), and confirmed or suspected aspirin intolerance or history of periodontal therapy in the last 12 months. Systemic inflammatory pathologies were ruled out using the Health Assessment Questionnaire (HAQ), endorsed and utilized by the Colombian Rheumatology Society^11^.

All participants were provided with written information regarding the study's benefits and potential risks, and their informed consent was obtained following the principles outlined in the Declaration of Helsinki. The study protocols received thorough review and approval from the Dentistry Faculty's Research Ethics and Methodology Committee at the Universidad Nacional de Colombia (Approval No. 09-18).

### Periodontal diagnosis

A complete periodontal examination, including probing depths, clinical attachment levels, bleeding on probing (BoP), and plaque index at six sites per tooth, was measured using a UNC15 periodontal probe (Hu-Friedy, Chicago, USA) by only one calibrated examiner. The subjects were divided into healthy and periodontitis. The diagnosis of periodontitis and healthy periodontium were adjusted to the American Academy of Periodontology criteria in 2017^12^.

### Obtaining peripheral blood mononuclear cells (PBMC)

8 ml of blood was collected from study participants in vacutainer tubes with heparin, which were centrifuged at 1400 rpm for 10 minutes (Beckman Coulter Allegra® X-15R Centrifuge, Indianapolis, USA) to separate the plasma from the cellular elements. Subsequently, peripheral blood mononuclear cells were separated by density gradient using Ficoll®-Hypaque (GE Healthcare Bio-Sciences Corp, Piscataway, NJ, USA) for 30 min at 1400 × g. The cells were washed and resuspended in RPMI medium supplemented with antibiotics, 20 mM HEPES, and 10% fetal bovine serum (Sigma-Aldrich, Darmstadt, Germany).

### Cell culture

The PBMCs were resuspended at a density of 1 × 10^6^ cells/ml in 24-well culture plates in the presence of phosphate buffer saline (control) and *P. gingivalis-*LPS (1µg / ml)^13^. The cells were cultured for 24 h at 37℃ in 5% CO_2_. Afterward, the supernatant was collected by centrifugation at 1400 rpm for 10 min and then stored at −80℃ until use.

### Quantification of RvE1

The amount of RvE1 produced in the culture supernatant was quantified by ELISA following the manufacturer’s instructions (MyBiosource MBS269927, San Diego, CA, USA). Briefly, the samples were added to the plate previously coated with capture antibody anti-resolvin E1. The wells were sealed and incubated at 37℃ for 90 min; then, the washing process with buffer solution was continued, and 100 μl of anti-resolvin E1 coupled to biotin (detection antibody) were added to each of the wells. These were sealed and incubated at 37℃ for 60 min; the wells were then immediately washed with a buffer solution and incubated with 100 μl of streptavidin peroxidase for 30 min at 37℃. Finally, the wells were washed, and the ELISA was performed by adding 100 µl of a previously prepared solution of TMB + hydrogen peroxide, and 100 µl of sulfuric acid was added to stop the reaction. The optical density of the wells was read in a spectrophotometer at a wavelength of 450 nm. A standard curve was used to interpolate the values (0.107-246 ng/mL). The detection limit of the assay was 0.06 ng/ml.

### Statistical analysis

A power analysis determined the minimum sample size required to detect a meaningful difference in resolvin levels between the study groups. A power of 80% was chosen, with a significance level (alpha) of 0.05. As there is no baseline information evaluating resolvin levels in periodontitis patients, we searched the literature to include studies quantifying resolvin levels in peripheral blood in any population or experimental conditions. Based on a previous study^10^ in healthy individuals, Resolvin E3 reached 154 ± 171 pg/mL in peripheral blood in response to fish oil intake, which was statistically different from the placebo group (32 ± 54 pg/mL, P = 0.04). Using http://biomath.info/power/prt.htm and assuming the mean difference and its standard deviation, the resulting sample size was 10-12 subjects to find such a difference. Statistical significance for clinical parameters was determined by t-test and chi-square with Fischer correction. For the Resolvin E1 analysis, parametric testing for normally distributed data (one-way ANOVA with Tukey's comparisons) using GraphPad Prism 10 software (GraphPad Software, La Jolla, CA, USA) was used. The graph represents the median of the experiments. *p* < 0.05 was considered statistically significant.

## Results

### Periodontal clinical parameters

Of the 20 participants evaluated, 14 were women with a mean age of 36.4 years, and 6 were men with a mean age of 39.7 years; they were matched by sex and age (+/− 2 years). All participants were systemically healthy based on questioning.

The group of patients with periodontitis consisted of 10 participants with a diagnosis of generalized stage III and stage IV periodontitis. The healthy group consisted of 10 patients with a periodontal diagnosis of healthy periodontium or biofilm-induced gingivitis (Table 1).

Clinical parameters were markedly different between the 2 groups, with a mean depth to periodontal probing of 2.1 ± 0.36mm in healthy patients and 4.98 ± 0.42mm in patients with periodontitis (p<0.0001). There was a greater presence of biofilm in the group with periodontitis compared to healthy controls, 85.14% vs 22.75% (p<0.0001), and a higher percentage of bleeding on probing in patients with periodontitis, 26.81% vs 73.58% (p<0.0001), which revealed an ongoing inflammatory process. The differences in sites with periodontal pockets of 4-5mm and those with pockets of ≥6mm, as well as CAL in all categories (1-2mm, 3-4mm, and ≥5mm), were statistically significant between the groups, always being higher in patients with periodontitis.

**Table 1:** Clinical parameters in the patients of the two study groups

Clinical Parameters	Healthy group	Periodontitis group	p Value
Number of teeth	26.7 ± 1.9	26.4 ± 2.6	p= 0.771*
Bleeding index (%)	26.81	73.58	p<0.0001**
Plaque index (%)	22.75	85.14	p<0.0001**
Probing depth (mm)	2.1 ± 0.36	4.98 ± 0.42	p<0.0001*
Probing depth 4-5mm	2 ± 2.5	49.8 ± 19.1	p<0.0001*
Probing depth ≥ 6mm	0	12.1 ± 11.9	p=0.0048*
Clinical attachment level (mm)	1.03 ± 0.31	3.21 ± 1.42	P=0.0002*
Clinical attachment level 1-2mm	138.4 ± 36.9	72.3 ± 40.8	p=0.0013*
Clinical attachment level 3-4mm	19.4 ± 28.2	51.1 ± 18.9	p= 0.0085*
Clinical attachment level ≥ 5mm	0.13 ± 0.35	35 ± 28.2	p=0.0010*

A t-test was used for the continuous variables * and chi-square and Fischer exact test were used to analyze categorical variables**. *p* < 0.05 was considered statistically significant.

### Production of Resolvin E1

**Figure 1:**
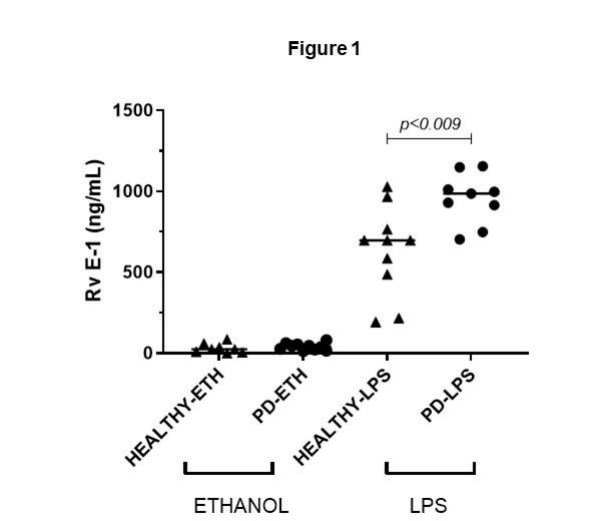
Effect of stimulation of peripheral blood mononuclear cells with *P. gingivalis*-LPS on RvE1 production. One-way ANOVA with Tukey's comparisons. The graph represents the median of the experiments. *p* < 0.05 was considered statistically significant.

To determine the effect of *P. gingivalis*-LPS on PBMC, the samples were stimulated for 24 h. Figure 1 shows the median values of RvE1 production by LPS-activated PBMC in healthy controls and patients with periodontitis. The results showed significantly higher production of RvE1 when PBMC was stimulated with LPS both in healthy controls and in patients with periodontitis compared to both the control groups with ethanol (*p* = 0.001). In the healthy group, a mean of 30.42 ng/ml (SD: 29.07) vs. 633.1 ng/ml (SD: 276.5) was observed without and with LPS activation, respectively; in patients with periodontitis, the mean was 40.1 ng/ml (SD: 21,6) vs. 1083.1 ng/ml (SD: 432.1) without and with LPS, respectively. Similarly, significantly higher production of RvE1 was observed in the group of patients with periodontitis compared to healthy patients in the presence of LPS (*p* = 0.009). (Figure 1)

There were no differences in RvE1 production by PBMCs non-LPS-activated between healthy and periodontitis patients.

## Discussion

The results of this study suggest a potential connection between systemic RvE1 activation and the potential protection from complications in other organs in the presence of chronic low-level systemic inflammation caused by a local periodontal infection. Additionally, the increased production of these agents may represent the body's efforts to mitigate the systemic effects of local infections through this mechanism.

The most significant finding was the marked increase in RvE1 production by PBMC in systemically healthy patients with periodontitis. This result suggests that when confronted with a known antigen, the immune system exhibits a pronounced predisposition to respond. Such a phenomenon within the innate immune system may be closely related to the concept of "trained immunity." Trained innate immunity, also known as innate immune memory, is the intriguing process in which cells of the innate immune system, including macrophages, monocytes, and natural killer cells, exhibit heightened and enduring reactivity to pathogens or microbial ligands following their initial encounter. This effect represents an initial yet long-lasting response, lasting from days to months. It is brought about by the reprogramming of various cells within the innate immune system, including endothelial cells, triggered by their activation in response to pathogens or microbial ligands. These reprogramming processes are mediated by epigenetic changes^14^ or metabolic modifications. Such modifications alter the response to future exposures, even to unrelated pathogens, in terms of both potency and magnitude, either by amplifying or suppressing the immune response^15^. The result is an enhanced resistance to infectious agents^16^. The innate immune system retains the ability to remember its prior exposure to exotoxins from *P. gingivalis*, allowing the host to respond in a sensitized but non-specific manner to potential reinfections.

In the model developed in this study, the peripheral blood mononuclear cells (PBMC) of patients with periodontitis exhibited higher production of the pro-resolution agent RvE1 when exposed to the LPS from *P. gingivalis*, compared to the healthy controls who had not previously encountered the exotoxin. This discovery, while acknowledging the study's limitations, suggests that the immune system could attempt to self-regulate the hyperactive inflammatory response by increasing the production of RvE1.

Understanding this response, characterized by increased production of regulatory agents, holds significant promise for the development of potential therapeutic strategies aimed at modulating both local and systemic pro-inflammatory responses that arise secondary to periodontal infections^8^ ^,^ ^17^. The mechanism by which pro-resolution agents exert their effects can be elucidated through the "class change" model of eicosanoid pathways in neutrophils^18^. In this model, there is an upregulation of 15-lipoxygenase by neutrophils during the late stages of inflammation, initiating a series of enzymatic reactions. It commences with the oxidation of arachidonic acid by lipoxygenase, resulting in the production of intermediates such as 5, 12, or 15-S-hydroxy-(p)-eicosatetraenoic acid. These intermediates are subsequently metabolized into lipoxins, including A4 and B4^19^. These lipoxins act as receptor agonists, stimulating the production of pro-resolution agents of inflammation, such as RvE1, contributing to the restoration of systemic homeostasis. This restoration process is facilitated by the recruitment of neutrophils to the site of inflammation and alterations in the phenotype of macrophages, which promote the phagocytosis of apoptotic neutrophils, consequently inhibiting the further secretion of pro-inflammatory cytokines ^4^ ^,^ ^18^ ^,^ ^19^ ^,^ ^20^. The enhancement of this mechanism through targeted modulation of the response may offer a valuable therapeutic alternative.

Conversely, the concept of trained innate immunity, aside from its critical role in responding to infections (including vaccine responses), has garnered significant attention in the context of chronic inflammatory and metabolic disorders ^16^ ^,^ ^21^ ^,^ ^22^ ^,^ ^23^. Thus, it becomes imperative to assess the potential systemic implications of this activation in response to localized infections like periodontitis, particularly in scenarios where bacteremia may result from periodontopathogenic infections. This assessment could shed light on the protective capacity of innate immunity against systemic infections originating from periodontal sources.

While the precise mechanisms responsible for orchestrating trained innate immunity within the periodontium remain somewhat elusive, there is evidence to suggest that an imbalanced response is associated with increased susceptibility to periodontal diseases^24^. Furthermore, responses occurring in the oral and other mucosal tissues can exert systemic effects, including potential links to autoimmunity^25^, as previously explored in our group's other publications. This mutual influence strongly implies that dysregulation at the systemic level is mirrored in the immune function of the oral cavity and vice versa.

Therefore, it is plausible to hypothesize that in response to local inflammation with systemic repercussions, the body must upregulate the production of pro-resolving factors, exemplified by RvE1 in our findings. This response may also involve increased production of regulatory and anti-inflammatory cytokines, such as IL-10, TGF-β, IL-4, IL-13, IL-22, and others. This mechanism is likely in place to maintain a delicate balance of responses, thereby exerting control over the progression of periodontal diseases and their potential impact on other organs within the human body that are susceptible to systemic chronic inflammatory conditions.

To the best of our knowledge, this preliminary report represents the first evidence of the potential role of systemic activation of pro-resolution mechanisms in response to plausible transient bacteremia occurring alongside localized periodontal infections.

## Conclusions

Periodontitis appears to influence the RvE1-mediated systemic resolution response triggered by *P.gingivalis*-LPS induction.
